# Biological behavior of familial papillary thyroid microcarcinoma: Spanish multicenter study

**DOI:** 10.1007/s00423-022-02704-4

**Published:** 2022-10-17

**Authors:** A. Ríos, M. A. Rodríguez, J. A. Puñal, P. Moreno, E. Mercader, E. Ferrero, J. Ruiz-Pardo, M. A. Morlán, J. Martín, M. Durán-Poveda, J. M. Bravo, D. Casanova, M. P. Salvador Egea, N. M. Torregrosa, A. Exposito-Rodríguez, G. Martínez-Fernández, A. M. Carrión, O. Vidal, F. Herrera, G. Ruiz-Merino, J. M. Rodríguez

**Affiliations:** 1 Unidad de Cirugía Endocrina, Servicio de Cirugía General Y de Aparato Digestivo, Instituto Murciano de Investigación Bio-Sanitaria (IMIB-Arrixaca), Hospital Clínico Universitario Virgen de La Arrixaca, Servicio Murciano de Salud, Murcia, Spain; 2 Departamento de Cirugía, Pediatría Obstetricia, Y Ginecología, Universidad de Murcia, Murcia, Spain; 3 Servicio de Cirugía General Y Aparato Digestivo, C.H.U, Santiago de Compostela, Spain; 4 Cirugía Endocrina, Hospital Universitario de Bellvitge, L´Hospitalet de Llobregat, Barcelona, Spain; 5 Sección de Cirugía Endocrino-Metabólica, Hospital General Universitario Gregorio Marañón, Madrid, Spain; 6 Servicio de Cirugía General, Aparato Digestivo Y Trasplante de Órganos Abdominales, Hospital Universitario, 12 de Octubre, Madrid, Spain; 7 Servicio de Cirugía General Y del Aparato Digestivo, Hospital Universitario Torrecárdenas, Almeria, Spain; 8 Servicio de Cirugía General Y del Aparato Digestivo, Hospital Virgen de La Salud, Toledo, Spain; 9 Servicio de Cirugía General Y Aparato Digestivo, Hospital Universitario Severo Ochoa, Leganés, Madrid, Spain; 10 Servicio de Cirugía General Y del Aparato Digestivo, Hospital Universitario Rey Juan Carlos. Móstoles, Madrid, Spain; 11 Facultad de Ciencias de La Salud, Universidad Rey Juan Carlos, Alcorcón, Madrid, Spain; 12 Servicio de Cirugía General Y del Aparato Digestivo, Hospital de La Princesa, Madrid, Spain; 13 Servicio de Cirugía General Y del Aparato Digestivo, Hospital Universitario Marqués de Valdecilla, Santander, Spain; 14 Servicio de Cirugía General Y Digestiva, Complejo Hospitalario de Navarra, Pamplona, Spain; 15 Servicio de Cirugía General Y del Aparato Digestivo, Hospital de Santa Lucia, Cartagena, Murcia Spain; 16 Servicio de Cirugía General Y del Aparato Digestivo, Hospital de Basurto, Bizkaia, Spain; 17 Unidad de Cirugía Endocrina, Servicio de Cirugía General (Hospital Universitario de Cruces), Barakaldo, Bizkaia Spain; 18 Servicio de Cirugía, Hospital General Universitario de Alicante, Alicante, Spain; 19 Cirugía General Y del Aparato Digestivo, Hospital Universitario de Burgos, Burgos, Spain; 20 Servicio de Cirugía General, Hospital General Básico Santa Ana, Motril, Granada, Spain; 21 FFIS, Fundación Para La Formación E Investigación Sanitarias de La Región de Murcia, Murcia, Spain

**Keywords:** Papillary thyroid carcinoma, Familial papillary thyroid carcinoma, Papillary thyroid microcarcinoma, Familial papillary microcarcinoma, Recurrence

## Abstract

**Purpose:**

Familial papillary thyroid microcarcinoma (FPTMC) can present a more aggressive behavior than the sporadic microcarcinoma. However, few studies have analyzed this situation. The objective is to analyze the recurrence rate of FPTMC and the prognostic factors which determine that recurrence in Spain.

**Methods:**

Spanish multicenter longitudinal analytical observational study was conducted. Patients with FPTMC received treatment with curative intent and presented cure criteria 6 months after treatment. Recurrence rate and disease-free survival (DFS) were analyzed. Two groups were analyzed: group A (no tumor recurrence) vs. group B (tumor recurrence).

**Results:**

Ninety-four patients were analyzed. During a mean follow-up of 73.3 ± 59.3 months, 13 recurrences of FPTMC (13.83%) were detected and mean DFS was 207.9 ± 11.5 months. There were multifocality in 56%, bilateral thyroid involvement in 30%, and vascular invasion in 7.5%; that is to say, they are tumors with histological factors of poor prognosis in a high percentage of cases. The main risk factors for recurrence obtained in the multivariate analysis were the tumor size (OR: 2.574, 95% CI 1.210–5.473; *p* = 0.014) and the assessment of the risk of recurrence of the American Thyroid Association (ATA), both intermediate risk versus low risk (OR: 125, 95% CI 10.638–1000; *p* < 0.001) and high risk versus low risk (OR: 45.454, 95% CI 5.405–333.333; *p* < 0.001).

**Conclusion:**

FPTMC has a recurrence rate higher than sporadic cases. Poor prognosis is mainly associated with the tumor size and the risk of recurrence of the ATA.

**Graphical abstract:**

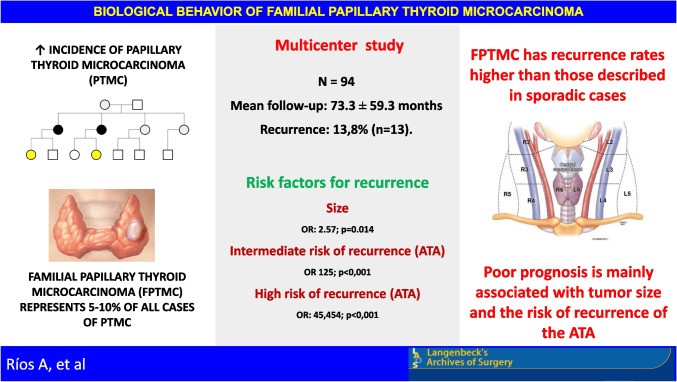

## Introduction

The incidence of papillary thyroid microcarcinoma (PTMC) has increased in recent decades. This is largely due to the high-resolution ultrasonography and thyroid pathology screening in many countries [1]. Since this tumor generally has an excellent prognosis, several groups, especially in Asia, have proposed conservative treatment with active surveillance [2, 3].

Familial papillary thyroid microcarcinoma (FPTMC) accounts for approximately 5 to 10% of all diagnosed cases of PTMC [4–6]. However, its frequency is increasing due to screening programs in families with familial papillary thyroid carcinoma (FPTC) [4].

While sporadic papillary thyroid microcarcinoma (SPTMC) has a favorable prognosis, the biological behavior of FPTMC is little known and could have a probably more aggressive course [6, 7]. In this sense, it is described that FPTMC can present a high rate of multifocality, vascular involvement, and lymph node metastasis [8–12]. However, there are few and short series in order to recommend a more aggressive treatment [6, 7, 9–12].

There are currently only 5 studies that analyze FPTMC [6, 7, 9–11], and two of them have a high risk of bias according to the Newcastle–Ottawa scale [7, 10]. Furthermore, some case series are very small, such as that of Lupoli G. et al. [7] with only 7 cases and Capezzone M. et al. [11] with 43 cases. On the other hand, the most numerous series have a very short follow-up. In this sense, the series of 217 cases presented by Cao J. et al. [10] has only a mean follow-up of 34 months.

Despite this lack of consistent evidence, there are guidelines such as the 2015 American Thyroid Association (ATA) that recommends a more aggressive management of the FPTC [13]. For this reason, active surveillance is not considered in FPTMC [2, 3] and the minimal surgery that is recommended is total thyroidectomy. Even authors such as Lee et al. [6] recommend associating a central neck dissection.

The objective of this study is to analyze the recurrence rate of FPTMC and the factors related to recurrence in a national multicenter study.

## Material and methods

### Type of study

National multicenter longitudinal analytical observational study was conducted by surgeons of the Endocrine Surgery Section of the Spanish Society of Surgery.

### Study population

The study population consisted of patients diagnosed with FPTMC. This tumor was defined as the presence of a papillary thyroid carcinoma (PTC) equal to or less than 1 cm in size in a family diagnosed with FPTC.

FPTC was defined as the presence of at least two first-degree relatives with a histologically confirmed PTC. Patients with any of the following criteria were excluded from this concept: families with multiple endocrine neoplasia (MEN) syndrome, families with Cowden syndrome, families with Gardner syndrome, familial adenomatous polyposis, Carney complex, or people with previous exposure to ionizing radiation.

Patients with FPTMC who met the following criteria were included in this study:

- To receive curative intent treatment.
- To meet cure criteria 6 months after definitive treatment.
- One-year minimum follow-up.

Patients with FPTMC who met the following criteria were excluded from this study:

- Persistent disease after treatment.
- Breach by the patient of the therapeutic and follow-up protocol.
- Incomplete medical history and inability to complete it.
- Follow-up less than 1 year.

### Preparation of the data collection protocol

A data collection protocol was developed by the project manager, which was approved by the Endocrine Surgery Section of Spanish Society of Surgery. The study protocol was approved by institute’s committee (2021–2-13-HCUVA).

A first direct contact was made by the project manager with the different Endocrine Surgery Units to explain the project, and later an institutional contact through the secretariat of the Spanish Society of Surgery to provide the documentation in order to participate in the project.

### Evaluation and approval of the cases under study

The project was developed over a period of 4 years (2015–2018). Subsequently, the follow-up was updated but without including more patients. The information provided for each case was the complete protocol and the family tree that confirmed that it corresponded to a FPTC. The information was obtained from the patients and from the family history of the patients.

All cases were evaluated by the same investigator to confirm the validity of each case. Once its validity is accepted, it is formalized and any doubt or contradiction regarding the case was consulted to resolve it.

### Study groups

The patients included in the study were divided into two groups according to the presence or absence of recurrent disease during the follow-up. Recurrence was defined as the presence of clinical, radiological, or biochemical (increased serum thyroglobulin with negative antithyroglobulin antibodies) disease after 6 months of complete radical treatment.

The two groups under study were.

- Group A: No FPTMC recurrence during follow-up.
- Group B: Presence of FPTMC recurrence during follow-up.

### Study variables

To assess potential risk factors, the following groups of variables were analyzed:

1. Socio-familial variables: age, gender, and number of cases of FPTMC in the family.
2. Clinical variables: asymptomatic, cervical tumor, dysphonia, dysphagia, and dyspnea.
3. Thyroid function: euthyroidism, hypothyroidism, and hyperthyroidism.
4. Histological variables: histological variant, tumor size (in millimeters), multifocality, number of foci, bilaterality, vascular invasion, lymphatic involvement, and chronic lymphocytic thyroiditis.
5. Tumor stage according to the 7th and 8th editions of the TNM Staging System of the American Joint Committee on Cancer (AJCC).
6. Assessment of risk of recurrence according to the 2015 ATA guidelines.

### Statistical analysis

The data were analyzed using the statistical program SPSS® v21.0 for Windows® (SPSS, Chicago, IL, USA). For bivariate analysis of the different variables as risk factors, a Cox analysis was performed. Multivariate analysis was performed using a binary logistic regression to evaluate the independent associations of all factors that were statistically significant in the bivariate analysis. The results were expressed as odds ratio (OR) with a 95% confidence interval (95% CI) and *p* value.

The Kaplan–Meier method was used to analyze the disease-free survival (DFS) and the Log Rank test to compare survival between groups. A *p* value < 0.05 was considered statistically significant.

## Results

### Series description

Of the 30 Spanish Endocrine Surgery Units that indicated their commitment to participate in the project, 20 of them detected cases of FPTMC in their centers and were able to participate in the project.

Ninety-four patients of 60 families with FPTC met the inclusion criteria in the project. Surgery was performed in all cases: total thyroidectomy in 89% (*n* = 84) and lobectomy in 11% (*n* = 10). Cervical lymph node dissection was associated in 30% (*n* = 28): central neck dissection in 28 cases (unilateral in 17 cases [18%] and bilateral in 11 cases [12%]) and lateral neck dissection in 7 cases (ipsilateral in 4 and bilateral in 3 cases). Radioactive iodine (I^131^) was administered in 86% of the patients (*n* = 81).

**Fig. 1:**
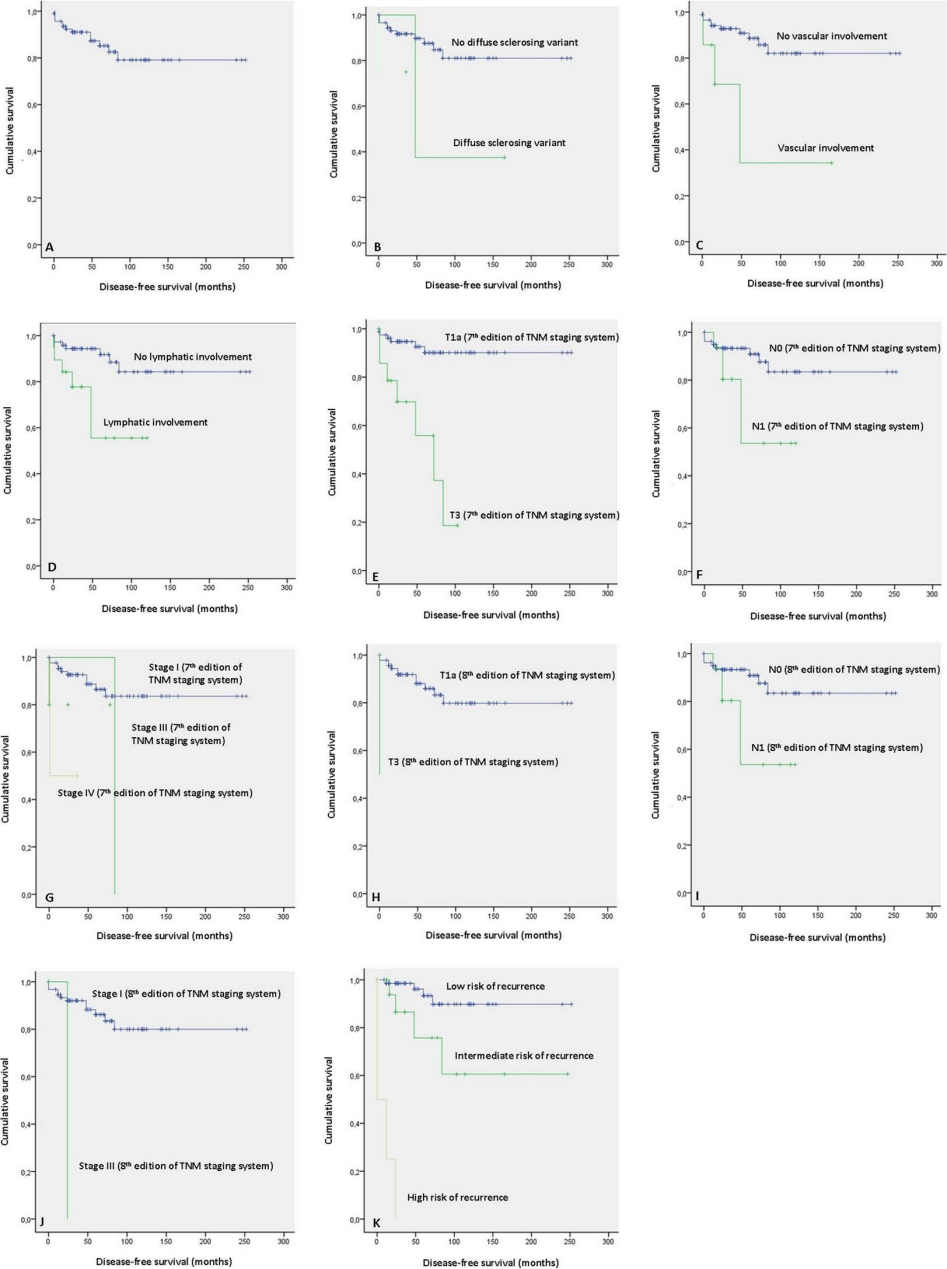
Disease-free survival (DFS) of familial papillary thyroid microcarcinoma. **A** Mean DFS. **B** DFS according to the diffuse sclerosing variant. **C** DFS according to the vascular invasion. **D** DFS according to the lymphatic involvement. **E** DFS according to “T” of the 7th edition of the TNM Staging System of the American Joint Committee on Cancer. **F** DFS according to “N” of the 7th edition of the TNM Staging System of the American Joint Committee on Cancer. **G** DFS according to the tumor stage of the 7th edition of the TNM Staging System of the American Joint Committee on Cancer. **H** DFS according to “T” of the 8th edition of the TNM Staging System of the American Joint Committee on Cancer. **I** DFS according to “N” of the 8th edition of the TNM Staging System of the American Joint Committee on Cancer. **J** DFS according to the tumor stage of the 8th edition of the TNM Staging System of the American Joint Committee on Cancer. **K** DFS according to the assessment of risk of recurrence of the American Thyroid Association

During a mean follow-up of 73.3 ± 59.3 months, 13 recurrences (13.8%) were detected. The mean DFS was 207.9 ± 11.5 months (Fig. 1).

### Socio-family and clinical factors associated with tumor recurrence

No association was observed between socio-familial or clinical factors and the presence of FPTMC recurrence (Table 1).

**Table 1:** Socio-familial and clinical factors associated with the recurrence of familial papillary thyroid microcarcinoma: Cox and survival analyses

Variables	Standard error	Odds ratio	95% confidence interval	*P*	DFS 5 years	DFS 10 years	Mean DFS (months)	*P*
	Cox analysis				Survival analysis			
Socio-familial factors								
Age (40.76 ± 11.01 years)	0.026	0.989	0.939–1.041	0.668				
Age (< 45 years and ≥ 45 years)								
< 45 years (*n* = 58; 8 recurrences) ≥ 45 years (*n* = 36; 5 recurrences)	0.572	1 0.922	0.301–2.827	0.887	84% 86.6%	79.3% 78.7%	203.54 ± 14.45 208.28 ± 18.42	0.887
Age (< 55 years and ≥ 55 years)								
< 55 years (*n* = 83; 12 recurrences) ≥ 55 years (*n* = 11; 1 recurrence)	1.042	1 0.569	0.074–4.393	0.589	84.3% 90%	77.1% 90%	204.36 ± 12.97 216.1 ± 22.67	0.583
Gender								
Male (*n* = 22; 1 recurrence) Female (*n* = 72; 12 recurrences)	1.041	1 0.262	0.034–2.019	0.199	82.6% 94.7%	74.5% 94.7%	194.86 ± 13.95 239.58 ± 12.09	0.165
Number of cases of PTC in the family								
Families with 2 (*n* = 37; 5 recurrences) Families with > 2 (*n* = 57; 8 recurrences)	0.571	1 1.031	0.337–3.156	0.958	81% 87.7%	81% 77.5%	131.2 ± 9.41 205.61 ± 15.64	0.957
Number of cases of PTC in the family								
Families with 2 (*n* = 37; 5 recurrences) Families with 3 (*n* = 23; 2 recurrences) Families with > 3 (*n* = 34; 6 recurrences)	0.592 0.318	1 1.030 1.124	0.357–3.177 0.603–2.093	0.923 0.714	81% 94.4% 83.3%	81% 78.7% 75.7%	131.2 ± 9.41 142.08 ± 15.18 200.06 ± 19.4	0.755
Clinical factors								
Asymptomatic								
Yes (*n* = 79; 11 recurrences) No (*n* = 15; 2 recurrences)	0.769	1 1.159	0.257–5.230	0.848	100% 82.3%	62.5% 82.3%	187 ± 36.87 207.87 ± 10.97	0.847
Cervical tumor								
No (*n* = 46; 7 recurrences) Yes (*n* = 48; 6 recurrences)	0.558	1 0.709	0.237–2.117	0.538	88.5% 83.3%	72.6% 83.3%	133.28 ± 11.19 215.73 ± 13.73	0.534
Dysphonia								
No (*n* = 93; 13 recurrences) Yes (*n* = 1; 0 recurrence)	14.716	1 0.049	0–1.643E^11^	0.838	85.1% 100%	79% 100%	207.96 ± 11.56 ∞	0.758
Dysphagia								
No (*n* = 93; 13 recurrences) Yes (*n* = 1; 0 recurrence)	14.716	1 0.049	0–1.643E^11^	0.838	85.1% 100%	79% 100%	207.96 ± 11.56 ∞	0.758
Dyspnea								
No (*n* = 91; 13 recurrences) Yes (*n* = 3; 0 recurrence)	7.244	1 0.047	0–69.479017	0.674	84.8% 100%	78.5% 100%	207.96 ± 11.56 ∞	0.524

DFS: disease-free survival

### Histological factors associated with tumor recurrence

Regarding the histological variant, only the diffuse sclerosis variant was associated with a higher recurrence rate, with an OR of 4.869 (*p* = 0.040) and a DFS at 5 years of 37.5% (compared to 87, 5% of the rest of the variants; *p* = 0.022) (Table 2).

**Table 2:** Association of the thyroid function and the histological variants with the recurrence of the familial papillary thyroid microcarcinoma: Cox and survival analyses

Variables	Standard error	Odds ratio	95% confidence interval	*P*	DFS 5 years	DFS 10 years	Mean DFS (months)	*P*
	Cox analysis				Survival analysis			
Thyroid function								
Euthyroidism (*n* = 63; 9 recurrences) Hyperthyroidism (*n* = 8; 2 recurrences) Hypothyroidism (*n* = 23; 2 recurrences)	0.782 1.004	1 1.515 2.327	0.327–7.022 0.325–16.650	0.282 0.708	82.5% 100% 86.8%	82.5% 50% 86.8%	212.32 ± 12.37 162.5 ± 42.30 147.39 ± 11.84	0.693
Histological variant^*^								
Conventional/classical variant								
No (n = 32; 4 recurrences) Yes (n = 62; 9 recurrences)	0.603	1 1.074	0.330–3.498	0.906	87.3% 84.1%	76.4% 79.4%	199.99 ± 22.62 207.98 ± 13.76	0.906
Follicular variant								
No (n = 72; 12 recurrences) Yes (n = 22; 1 recurrence)	1.044	1 0.306	0.040–2.369	0.257	81.3% 100%	77.6% 75%	203.21 ± 12.91 125.25 ± 15.37	0.228
Clear cell variant								
No (n = 90; 12 recurrences) Yes (n = 4; 1 recurrence)	1.043	1 1.522	0.197–11.749	0.687	85.7% 75%	79% 75%	208.25 ± 12.03 189.25 ± 50.01	0.684
Tall cell variant								
No (n = 92; 12 recurrences) Yes (n = 2; 1 recurrence)	1.043	1 3.417	0.442–26.415	0.239	86% 50%	79.7% 50%	209.5 ± 11.7 131.5 ± 81.67	0.209
Diffuse sclerosing variant								
No (n = 90; 11 recurrences) Yes (n = 4; 2 recurrences)	0.770	1 4.869	1.077–22.016	**0.040**	87.5% 37.5%	81% 37.5%	212.42 ± 11.49 79.88 ± 38.66	0.022
Mixed variant								
No (n = 92; 13 recurrences) Yes (n = 2; 0 recurrence)	8.277	1 0.048	0–532.334394	0.714	84.9% 100%	78.6% 100%	207.96 ± 11.56 ∞	0.048

DFS: disease-free survival

^*^The total number of patients is 94 and the total number of histological variants is 96. Two patients with multicentric tumors present two different histological variants in different foci

Bold are the p<.05

The association with tumor size stands out with an OR of 1.403 (*p* = 0.029), in such a way that as the tumor size increases by 1 mm, the probability of presenting a recurrence increases 1.403 times. Regarding the presence of vascular invasion OR was 5.302 (*p* = 0.013) and for the presence of lymphatic involvement OR was 4.263 (*p* = 0.010) (Table 3). The 5-year DFS of vascular invasion and lymphatic involvement were 34.3% (*p* = 0.005) and 55.5% (*p* = 0.005), respectively (Table 3; Fig. 1).

**Table 3:** Histological factors associated with the recurrence of familial papillary thyroid microcarcinoma: Cox and survival analyses

Variables	Standard error	Odds ratio	95% confidence interval	*P*	DFS 5 years	DFS 10 years	Mean DFS (months)	*P*
	Cox analysis				Survival analysis			
Tumor size	0.155	1.403	1.035–1.902	**0.029**				
Multifocality								
No (*n* = 41; 3 recurrences) Yes (*n* = 53; 10 recurrences)	0.660	1 3.042	0.834–11.099	0.092	88.9% 82.9%	88.9% 69.6%	229.05 ± 12.61 117.6 ± 9.22	0.075
Number of foci	0.178	1.239	0.874–1.756	0.229				
Bilaterality								
No (*n* = 66; 7 recurrences) Yes (*n* = 28; 6 recurrences)	0.557	1 0.505	0.169–1.503	0.219	82.2% 86.3%	64.1% 86.3%	114.82 ± 12.11 221.12 ± 11.16	0.208
Vascularinvolvement								
No (*n* = 87; 10 recurrences) Yes (*n* = 7; 3 recurrences)	0.668	1 5.302	1.432–19.632	**0.013**	88.6% 34.3%	82% 34.3%	214.76 ± 11.38 75.91 ± 33.63	**0.005**
Lymphatic involvement								
No (*n* = 75; 7 recurrences) Yes (*n* = 19; 6 recurrences)	0.560	1 4.263	1.422–12.781	**0.010**	91.8% 55.5%	84.3% 55.5%	220.27 ± 11.71 79.48 ± 13.03	**0.005**
Chronic lymphocytic thyroiditis								
No (*n* = 64; 11 recurrences) Yes (*n* = 30; 2 recurrences)	0.769	1 0.380	0.084–1.716	0.208	81.4% 92.9%	77.6% 81.3%	202.29 ± 13.75 208.18 ± 20.69	0.189

DFS: disease-free survival

Bold are the p<.05

### Tumor staging and tumor recurrence

#### Tumor staging according to the 7th edition of the TNM staging system of the AJCC

According to the 7th edition of the TNM Staging System of the AJCC, 84% of the cases (*n* = 79) were T1a and the remaining 16% (*n* = 15) were T3. T3 tumors have a higher recurrence rate (OR: 8.414; *p* < 0.001) and a shorter DFS than T1a (55.9% at 5 years; *p* < 0.001). Regarding the assessment of cervical lymph node involvement (N), 17% of the cases (*n* = 16) presented involvement (N1). The recurrence rate was higher in the group with lymph node involvement (OR: 3.413; *p* = 0.032) and presented a shorter DFS (Table 4; Fig. 1).

**Table 4:** Tumor staging factors associated with the recurrence of familial papillary thyroid microcarcinoma: Cox and survival analyses

Variables	Standard error	Odds ratio	95% confidence interval	*P*	DFS 5 years	DFS 10 years	Mean DFS (months)	*P*
	Cox analysis				Survival analysis			
Tumor staging according to the 7th edition of TNM staging system of the AJCC								
Tumor stage								
I (*n* = 87; 10 recurrences) III (*n* = 5; 2 recurrences) IV (*n* = 2; 1 recurrence)	1.080 1.259	1 9.434 2.045	1.133–76.923 0.173–23.809	**0.038** 0.570	86.5% 80% 50%	83.6% 0% 50%	216.82 ± 10.57 67.2 ± 21.25 18.5 ± 12.37	**0.011**
T								
T1a (*n* = 79; 6 recurrences) T3 (*n* = 15; 7 recurrences)	0.560	1 8.414	2.805–25.237	** < 0.001**	90.1% 55.9%	90.1% 18.6%	230.02 ± 8.7 57.97 ± 10.79	** < 0.001**
N								
N0 (*n* = 78; 8 recurrences) N1 (*n* = 16; 5 recurrences)	0.574	1 3.413	1.107–10.526	**0.032**	90.9% 53.6%	83.4% 53.6%	217.99 ± 11.77 81.11 ± 13.56	**0.022**
Tumor staging according to the 8th edition of TNM staging system of the AJCC								
Tumor stage								
I (*n* = 92; 12 recurrences) II (*n* = 2; 1 recurrence)	1.074	1 9.009	1.094–71.429	**0.041**	86.2% 0%	80% 0%	210.25 ± 11.46 24 ± 0	**0.012**
T								
T1a (*n* = 92; 12 recurrences) T3 (*n* = 2; 1 recurrence)	1.225	1 23.256	2.088–250	**0.010**	71.1% 0%	67.9% 0%	210.14 ± 11.47 12 ± 0	** < 0.001**
N								
N0 (*n* = 78; 8 recurrences) N1 (*n* = 16; 5 recurrences)	0.574	1 3.413	1.107–10.526	**0.032**	90.9% 53.6%	83.4% 53.6%	217.99 ± 11.77 81.11 ± 13.56	**0.022**
Risk of recurrence according to the ATA								
Low risk (*n* = 70; 4 recurrences) Intermediate risk (*n* = 18; 4 recurrences) High risk (*n* = 6; 5 recurrences)	0.853 0.840	1 111.111 23.809	20–500 4.608–125	** < 0.001** ** < 0.001**	93.4% 75.7% 0%	89.8% 60.6% 0%	231.83 ± 9.83 170.27 ± 31.75 9 ± 4.74	** < 0.001**

AJCC: American Joint Committee on Cancer, ATA: American Thyroid Association

Bold are the p<.05

Regarding the tumor staging, 92.6% (*n* = 87) were stage I, 5.3% (*n* = 5) were stage III, and the remaining 2.1% (*n* = 2) were stage IV. Stage I presented a lower recurrence rate and a higher DFS (Table 4; Fig. 1).

#### Tumor staging according to the 8th edition of the TNM staging system of the AJCC

According to the 8th edition of the TNM Staging System of the AJCC, 97.9% of the cases (*n* = 92) were T1a and the remaining 2.1% (*n* = 2) were T3. T3 cases presented a higher risk of recurrence (OR: 23.256; *p* = 0.010) and a shorter DFS than T1a (0% at 5 years; *p* < 0.001). Regarding the assessment of cervical lymph node involvement (N), 17% (*n* = 16) presented involvement (N1). The recurrence rate was higher in the group with lymph node involvement (OR: 3.413; *p* = 0.032) and presented a shorter DFS (Table 4).

Regarding the tumor staging, 97.9% (*n* = 92) corresponded to stage I and the rest to stage II. Stage II presented a higher risk of recurrence (OR: 9.009; *p* = 0.041) and a shorter DFS (Table 4; Fig. 1).

#### Assessment of the risk of recurrence according to the ATA

The assessment of the risk of recurrence according to the ATA had a strong association with recurrence. Thus, intermediate risk presented an OR of 111.111 compared to low risk (*p* < 0.001) and high risk presented an OR of 23.809 compared to low risk (*p* < 0.001) (Table 4; Fig. 1).

### Multivariate analysis

The risk factors included in the multivariate analysis were those that were significant in the bivariate analysis: diffuse sclerosing variant, tumor size, vascular invasion, lymphatic involvement, and the TNM staging system. Because both the 7th and 8th editions were used for TNM staging, it was decided to include the 7th edition since the 8th edition homogenizes the results (97.9% were stage I). The assessment of the risk of recurrence according to the ATA was not included because this classification groups include various risk factors.

The risk factors obtained were the diffuse sclerosing variant and the tumor size (Table 5). Thus, patients with diffuse sclerosing variant had an 18.7 times higher probability of recurrence (OR: 18.765; *p* = 0.034) than other variants. Regarding tumor size, an increment of 1 mm in size increased the probability of recurrence 1.8 times compared to the previous size (OR: 1.806; *p* = 0.011).

**Table 5:** Recurrence factors of familial papillary thyroid microcarcinoma: multivariate analysis

Variables	Regression coefficient (*β*)	Standard error	Wald statistic	Odds ratio	95% confidence interval	*P*
Not including the risk of recurrence according to the ATA						
Diffuse sclerosing variant						
No				1		
Yes	2.932	1.382	4.499	18.765	1.250–281.809	**0.034**
Tumor size	0.591	0.232	6.473	1.806	1.145–2.848	**0.011**
Including the risk of recurrence according to the ATA						
Tumor size	0.945	0.385	6.033	2.574	1.210–5.473	**0.014**
Risk of recurrence according to the ATA Low risk Intermediate risk High risk	4.770 3.811	1.227 1.083	15.108 12.377	1 125 45.454	10.638–1000 5.405–333.333	** < 0.001** ** < 0.001**

ATA: American Thyroid Association

Bold are the p<.05

A second multivariate analysis was performed, also including among the variables the risk of recurrence of the ATA. In this case, the tumor size and the assessment of the risk of recurrence of the ATA persisted as risk factors (Table 5). In this sense, an increment of 1 mm in tumor size increased the probability of recurrence 2.5 times compared to the previous size (OR: 2.574; *p* = 0.014). Regarding the risk of recurrence of the ATA, patients with an intermediate risk had a probability of recurrence 125 times higher than those with a low risk (OR: 125; *p* < 0.001), and patients with a high risk had a probability of recurrence 45.454 times higher than those with a low risk (OR: 45.454; *p* < 0.001) (Table 5).

## Discussion

PTMC is considered an indolent tumor because active surveillance achieves good results avoiding the morbidity associated with surgery [2, 3]. However, FPTMC appears to be more aggressive [4, 6–12]. On the other hand, since the outcomes are linked to an adequate initial treatment [14, 15], although the treatment could associate a greater morbidity, the more aggressive tumors should be treated more aggressively [14].

The recurrence rate of SPTMC is relatively low, below 4% in majority of studies and generally less than 2% [15, 16]. On the contrary, the reported recurrence rate in FPTMC is more variable. The first problem in this regard is the scarcity of studies reporting on this pathology, the second is the low number of patients included in these studies, and the third is that these studies are all retrospective [6–12]. In this manner, the recurrence rate is above 2%, ranging between 2.1% [9] and 42.8% [7], although the study that provides such a high rate analyzes only 7 patients. The Spanish multicenter study also shows a high recurrence rate for FPTMC, specifically13.8%. This is an important data taking into account the mean follow-up longer than 6 years. Therefore, despite the heterogeneity of the data, patients with FPTMC, unlike SPTMC [17, 18], should not be considered for conservative treatment like active surveillance due to the high recurrence rate [2, 3].

When risk factors for recurrence of FPTMC are analyzed, one of the main prognostic factors is tumor size, so for every millimeter the risk of recurrence is practically twofold compared to the previous size. Although it is difficult to establish a cut-off point, in our series a size less than 4–5 mm reduces the risk of recurrence. These small tumors are the most indolent and with best prognosis [19]. Another aspect already known is the presence of histological variants of PTC with a poor prognosis, such as diffuse sclerosing variant [20]. It has not been observed in published studies that FPTC presents a higher incidence of more aggressive histological variants compared to sporadic carcinoma [4, 8, 10]. Finally, it should be noted that the assessment of the risk of recurrence of the ATA is highly predictive of the risk of FPTMC recurrence [21].

A more in-depth analysis of the data presented in this Spanish national study above FPTMC shows a high percentage of cases with tumor multifocality (56%), bilaterality (30%), and vascular invasion (7.5%). In other words, FPTMC has histological factors of poor prognosis in a high percentage of cases, a fact already indicated by other authors [11, 12]. There are several studies that show the high frequency of multifocality and bilaterality of FPTMC; even the finding of a multifocal tumor should suggest a familial disease [22]. It is necessary to remember that multifocality leads to higher rates of persistent local disease, cervical lymph node metastasis, and distant metastases, circumstances that cause a worse evolution and an increase of morbimortality [23, 24].

A controversial issue is the change in the 8th edition of TNM staging system compared to the previous edition in order to assess recurrence and survival differences. The 8th edition seems more predictive of mortality than the 7th edition [25], but in pathologies such as PTMC the application of the 8th edition is limited because mortality is practically zero, so it would require studies with large sample size and a very long follow-up in order to show differences. Thus, in this multicenter national study, the 8th edition practically does not differentiate and more than 97% of the cases are T1a and stage I, with a recurrence rate higher than 13%, so in this tumor the application of the 7th edition of TNM staging system is more discriminating. However, only in studies with large sample size such as the one presented by Yang et al. [25], which includes 39,032 microcarcinomas from the “National Cancer Institute’s Surveillance, Epidemiology and End Results (SEER),” the 8th edition has been shown to be more useful in order to assess survival. In our series, as it shows in Table 4, the application of the 8th edition is very homogeneous and does not allow defining the patients according to the recurrence and survival. For this reason, it is very difficult to perform a survival analysis in a relatively rare disease with a low mortality rate, and therefore, no study will probably be able to demonstrate differences in overall survival. On the contrary, DFS is a much more suitable variable to study diseases with these characteristics [26].

In conclusion, we can say that FPTMC is associated with a high recurrence rate, specifically higher than 13%, and presents poor prognostic histological parameters such as multifocality and vascular invasion. Although there are no studies that demonstrate the existence of a treatment more effective for FPTMC than for SPTMC, and therefore there is no evidence to support a more aggressive treatment, FPTMC should not be included in active surveillance protocols and the treatment should be surgical.

## Original report

This material has not been previously published or submitted elsewhere for publication and will not be sent to another journal until a decision is made concerning publication by Surgery.

## Data Availability

All data generated or analyzed during this study are included in this published article. The data underlying this article will be shared on reasonable request to the corresponding author (Dr. Ríos).

## Abbreviations

95% CI: 95% Confidence interval
AJCC: American Joint Committee on Cancer
ATA: American Thyroid Association
DFS: Disease-free survival
FPTC: Familial papillary thyroid carcinoma
FPTMC: Familial papillary thyroid microcarcinoma
OR: Odds ratio
PTC: Papillary thyroid carcinoma
PTMC: Papillary thyroid microcarcinoma
SPTMC: Sporadic papillary thyroid microcarcinoma
